# Impact of uncontrolled hypertension on 12-month clinical outcomes following below-the-knee arteries (BTK) interventions in patients with critical limb ischemia

**DOI:** 10.1186/s40885-016-0044-y

**Published:** 2016-02-29

**Authors:** Sung Il Im, Seung-Woon Rha, Byoung Geol Choi, Se Yeon Choi, Jae Joong Lee, Sun ki Lee, Ji Bak Kim, Jin Oh Na, Cheol Ung Choi, Hong Euy Lim, Jin Won Kim, Eung Ju Kim, Chang Gyu Park, Hong Seog Seo, Dong Joo Oh

**Affiliations:** 1 Division of Cardiology, Department of Internal Medicine, Kosin University Gospel Hospital, Busan, South Korea; 2 Cardiovascular Center, Korea University Guro Hospital, 80, Guro-dong, Guro-gu, Seoul 152-703 South Korea

**Keywords:** Uncontrolled hypertension, Below-the-knee artery (BTK) lesion, Peripheral angioplasty

## Abstract

**Background:**

Despite intensive anti-hypertensive treatment, overall control rates of only 30 ~ 50 % have been reported in patients with hypertension (HTN). However, clinical significance and angiographic characteristics of patients with uncontrolled HTN following Below-the-knee arteries (BTK) interventions in patients with critical limb ischemia (CLI) are not clarified yet as compared to those with controlled HTN.

**Methods:**

A total 165 consecutive hypertensive patients with BTK lesions from August 2004 to November 2012 were enrolled for this study. Uncontrolled HTN was defined as a blood pressure of > 140 mmHg systolic and 90 mmHg diastolic under anti-hypertensive treatment. A total of 112 patients (67.8 %) had uncontrolled HTN. We compared the clinical and angiographic characteristics of patients with uncontrolled HTN following BTK interventions to those with controlled HTN at 12-month follow-up.

**Results:**

The baseline characteristics are well balanced between the two groups. At 12 months, there was no difference in the incidence of mortality, target lesion revascularization (TLR), target extremity revascularization (TER), and limb salvage rate in both groups. However, amputation rates were higher in patients with controlled HTN (33.9 vs. 19.6 %, *P* = 0.045).

**Conclusion:**

Regardless of blood pressure control, HTN itself was an independent risk factor for BTK lesions, suggesting more intensive medical therapy with close clinical follow up will be required for all BTK patients with HTN.

## Background

Hypertension (HTN) is probably the most common risk factor of atherosclerotic cardiovascular disease. Atherosclerotic cardiovascular disease is the leading cause of mortality in Western countries [1]. Peripheral arterial disease (PAD), which is usually defined as atherosclerotic occlusion of the arterial bed in the lower extremities, is a major manifestation of systemic atherosclerosis. It is well known that PAD is associated with increased risk of mortality from cardiovascular disease (CVD) and all causes such as cerebrovascular diseases, renal disease, and diabetes mellitus (DM). In addition, it is an independent risk factor for mortality and morbidity in patients with CVD [2–4]. Therefore, the clinical importance of PAD has increasingly been acknowledged in recent several years [5].

Critical limb ischemia (CLI), characterized by ischemic rest pain or tissue loss, represent the most advanced state of PAD, burdened by high morbidity and mortality. CLI generally occurs in high risk patients with several risk factors including DM, older age, HTN with extensive atherosclerotic disease of below-the-knee vessels. Previous study also showed that PAD is significantly associated with systolic hypertension in the high risk group [6].

HTN currently affects 25 % of adults and may affect > 90 % of individuals during their lifetimes [7]. Therefore adequate control of blood pressure is of public health importance. However, recent studies indicated that 30 ~ 50 % of those with HTN are either untreated or under-treated [8]. No previous study of PAD has been performed in patients with uncontrolled HTN. Clinical significance and angiographic characteristics of patients with uncontrolled HTN following Below-the-knee arteries (BTK) interventions in patients with CLI are not clarified yet as compared to those with controlled HTN.

In this study, we sought to clarify the impact of uncontrolled HTN on clinical outcomes in patients with CLI following BTK interventions during 12 months follow-up.

## Methods

### Study population

We performed peripheral angiography (PAG) in 180 consecutive patients (male 76.7 %, mean age 67.1 ± 10.9 years) who had typical or atypical claudication or wound to confirm significant PAD at cardiovascular center in Korea University Guro Hospital, Seoul, South Korea.

From August 2004 to November 2012, all consecutive hypertensive patients with CLI undergoing angioplasty of at least 1 BTK vessel at our center were screened for enrollment. Inclusion criteria were the presence of hypertension, CLI (Rutherford class 4 or greater), stenosis or occlusion ≥ 40 mm of at least 1 tibial vessel with distal run-off to the foot, and agreement to 12-month angiographic evaluation.

The patients were excluded if they had one of the following conditions including advanced heart failure (New York Heart Association class III or IV), serum creatinine ≥ 3 mg/dL, life expectancy < 1 year, contraindication to combined antiplatelet treatment, planned major amputation before angiography because these conditions can be major causes of adverse cardiovascular events and could serve as the bias of PAD and the patients without HTN.

**Fig. 1:**
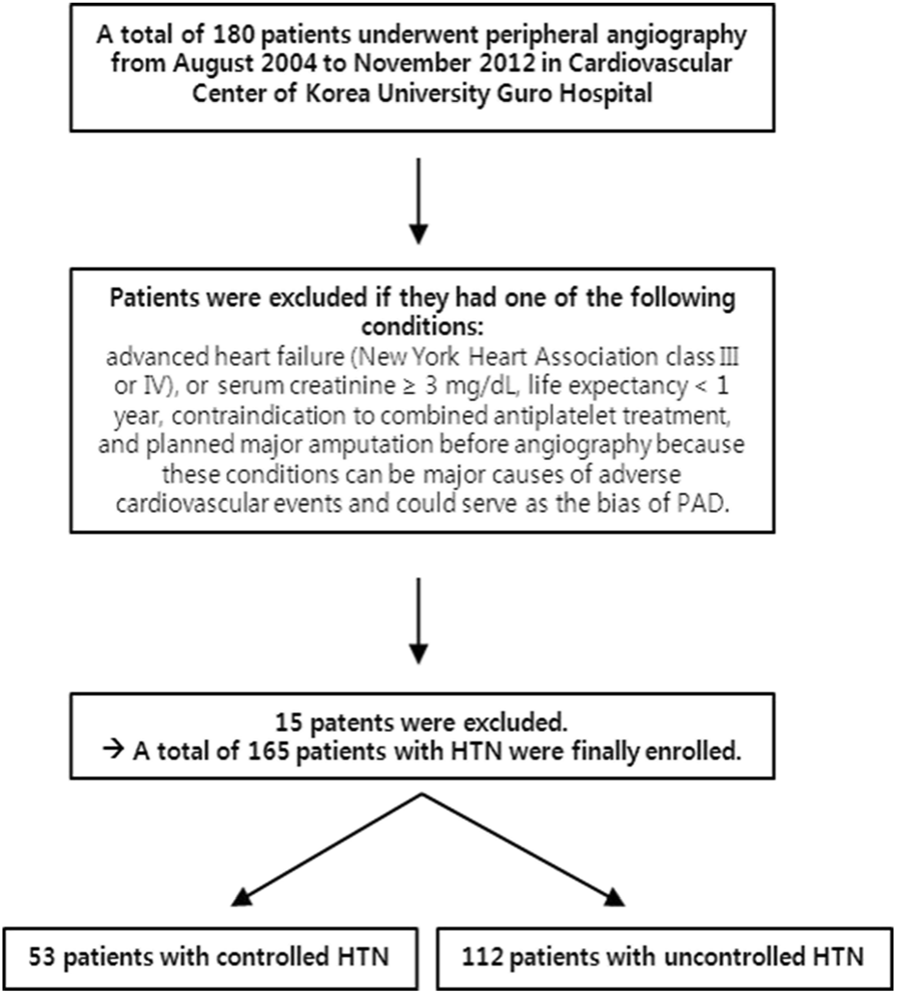
Flow chart

Finally, a total 165 hypertensive patients (91.6 % of total subjects underwent PAG) with angiographic proven PAD were enrolled for this study and underwent peripheral angioplasty. Those patients were divided into two groups according to blood pressure (BP) control (controlled HTN group; *n* = 53 patients, uncontrolled HTN group; *n* = 112 patients) and analyzed. The study flow chart was shown in Fig. 1. We compared the clinical and angiographic characteristics, and major clinical outcomes after BTK interventions up to 12 months in patients with HTN according to presence of BP control.

### Study definition

Hypertension was defined as either systolic or diastolic elevation of blood pressure ≥ 140/90 mmHg or ongoing antihypertensive pharmacological treatment. The physician measured blood pressure with sphygmomanometer with patients in a sitting posture, after resting for at least 5 min with the cuff placed on the arm. In each patient, the mean of two readings taken at intervals of at least 2 min was used in the study. The blood pressures were measured for all patients every three months from the enrollment. Uncontrolled HTN was defined as a blood pressure of > 140 mmHg systolic and 90 mmHg diastolic under anti-hypertensive treatment including patients who lack blood pressure control secondary to poor adherence and/or an inadequate treatment regimen, as well as those with true treatment resistance. Dyslipidemia was defined as a total cholesterol level ≥ 200 mg/dL or current treatment with lipid-lowering drugs. Current smoking was defined as active smoking within the past 12 months. Diabetes mellitus (DM) was defined as the fasting blood glucose level ≥ 126 mg/dL, or use of oral hypoglycemic agents or insulin. In the present study, if a patient’s past history, medical records, present symptoms or medical examination results accorded with one of the following criteria, the patients were diagnosed with PAD: (1) Claudication with ankle brachial indices < 0.90; (2) Claudication with findings of a significant lesion (≥70 % diameter stenosis) in peripheral artery on computed tomographic angiography (CTA) or invasive angiography; (3) Symptomatic carotid, subclavian arterial disease (≥70 % diameter stenosis) documented by image studies including CTA or invasive angiography. Insignificant CAD was defined as the ≤30 % diameter stenosis in peripheral arteries documented by image studies including CTA or invasive angiography.

### Study procedure

After admission, the femoral, popliteal, dorsalis pedis and posterior tibial arteries were palpated, and the extent of tissue loss was recorded as part of the pre-procedural study. Angioplasties were performed with crossover approach or an anterograde ipsilateral approach using 5 ~ 6 French sheaths. In case of failure to recanalize by either intraluminal or subintimal approach, a retrograde approach was attempted. After placing the sheath, intra-arterial heparin (70 IU/kg heparin) was routinely administered via the vascular sheath. A 0.014-in. guide wire was advanced into the lesion and a balloon catheter of optimal size was introduced. The appropriate balloon length and diameter were determined by visual assessment. Balloon inflation with normal pressure was maintained for at least 120 s. All patients were taking aspirin 100 mg daily at least 1 week prior to peripheral angioplasty. Post-intervention dual antiplatelet therapy with aspirin 100 mg and clopidogrel 75 mg once daily or additional cilostazol 100 mg twice a day were given at least for 4 weeks, and 100 mg aspirin was given daily thereafter.

Technical success was defined as restoration of direct flow in the target vessel with run-off to the foot and a residual stenosis < 30 %. Clinical success was defined as technical success without clinical events during hospitalization. In patients with bilateral CLI, an additional procedure for the revascularization of the contralateral limb was planned in a different session to limit the risk of x-ray exposure and contrast induced nephropathy, maintaining the same randomization arm.

### Follow-up

Once discharged, patients were followed up in Korea University Guro Hospital. Office visits were scheduled every two weeks for first 2 months, once a month for the third month, and then every 3 months. Minor amputations planned before the interventions were performed 2 to 4 weeks after revascularization an included toe amputations resulting from necrosis or infection of tissues and bones with preservation of healthy surrounding tissue. All patients were scheduled to be readmitted for control peripheral angiography at 12 months. In case of clinical CLI recurrence, angiography and repeat revascularization were performed within 1 week from diagnosis. In patients undergoing clinically driven repeat angiography of the target limb between 9 and 12 months who did not show evidence of restenosis of the target lesion, scheduled angiography at 12 months was not performed.

### Study End points and definitions

Before the intervention, immediately after the intervention, and at follow-up, angiography of the target vessel was performed in identical projections. The target lesion was identified by an image of the vascular anatomy and specific landmarks (collaterals, bone landmarks), with a second image showing the inflated balloons. These images were compared with follow-up angiograms.

The primary end point of the study was the comparison of the 12-month binary restenosis rates according to BP control. Restenosis was defined by angiography as a reduction in the luminal diameter > 50 % according to the worst angiographic view within the treated lesion plus the 10-mm segments proximal and distal to it.

The pre-specified secondary end points of the study were (1) clinically driven target lesion revascularization (TLR) defined as repeat percutaneous intervention or surgical bypass graft resulting from angiographic evidence of restenosis at the level of the treated lesion ±10 mm in the presence of at least 1 of the following criteria: recurrence of pain in the foot at rest that increased in the supine position, recurrence of foot lesion or evidence during follow-up of foot lesion size decrease–increase behavior or appearance of a new foot lesion; (2) major amputation, defined as unplanned amputation of the target limb in which a prosthesis was required for standing or walking, however, if the patients who refused the amputation, those patients were excluded from the analysis to reduce bias; and (3) target vessel occlusion (by CTA or invasive angiography). Acquired angiograms were reviewed by 2 blinded investigators who did not actively participate in recruitment and had no knowledge of clinical status and randomization group.

Multivariate analyses was performed for the cumulative 12-month prevalence of major adverse limb events (MALE) including total death, major amputations, TLR, and repeat BTK interventions in both groups.

### Statistical analysis

Data were analyzed according to the established standards descriptive statistics. Results were presented as numbers (percentages) of patients or medians (inter-quartile range) where applicable. Differences between groups stratified by blood control status in patients with HTN under anti-hypertensive medications were tested by x^2^ test and the Fisher’s exact test for dichotomous variables and the Mann–Whitney U test for continuous variables. Then, differences in clinical outcome between controlled HTN and Uncontrolled HTN groups were assessed at 12-months follow-up. Results were adjusted for age, gender and DM by logistic regression analysis. The difference in MALE between two groups during follow-up period was assessed by the Kaplan-Meier method by means of the log-rank test. All tests were 2-tailed and a *p* value of <0.05 was considered statistically significant. All statistical analysis was performed by means of SPSS 18.0 (SPSS Inc., Chicago, Illinois).

## Results

The baseline clinical characteristics and laboratory findings of patients are shown in Table 1. The most of variables including age, body mass index, dyslipidemia, smoking history, chronic kidney disease, congestive heart failure, and coronary artery disease were balanced between the two groups. There was no difference in baseline laboratory findings between the two groups.

**Table 1:** Baseline characteristics according to blood pressure control

Variable. n%	Total (*n* = 165 Pts) (*n* = 201 Limb) (*n* = 246 Lesion)	Controlled HTN (*n* = 53 Pts) (*n* = 66 Limb) (*n* = 90 Lesion)	Uncontrolled HTN (*n* = 112 Pts) (*n* = 135 Limb) (*n* = 156 Lesion)	*P* Value
Baseline characteristics				
Gender (Male)	128 (77.5)	43 (81.1)	85 (75.8)	0.550
Age (years)	68.2 ± 9.3	68.8 ± 10.2	67.9 ± 8.8	0.549
Body mass index (kg/m^2^)	23.2 ± 3.2	22.9 ± 3.4	23.3 ± 3.1	0.471
Diagnosis				
Wound	135 (81.8)	44 (83)	91 (81.2)	0.783
Diabetic foot ulcer	124 (75.1)	41 (77.3)	83 (74.1)	0.652
Gangrene	11 (6.6)	3 (5.6)	8 (7.1)	1.000
Claudication	12 (7.2)	5 (9.4)	7 (6.2)	0.525
Resting pain	16 (9.6)	3 (5.6)	13 (11.6)	0.228
Blood pressure; BP (mmHg)				
Systolic BP	151.7 ± 17.3	122.6 ± 13.2	165.4 ± 19.2	<0.001
Diastolic BP	76.2 ± 45.5	64.9 ± 12.4	81.5 ± 61.2	0.053
Heart rate	78.9 ± 14.7	79.1 ± 19.5	78.8 ± 12.4	0.911
Pulse pressure (mmHg)	78.6 ± 24.2	57.7 ± 0.8	83.8 ± 42.0	<0.001
Past medical and social history				
Known HTN	116 (70.6)	35 (66.2)	81 (73.2)	0.399
Diabetes	152 (92.1)	49 (92.4)	103 (91.9)	1.000
Dyslipidemia	8 (4.8)	3 (5.6)	5 (4.4)	0.713
Cerebrovascular disease	32 (19.3)	9 (16.9)	23 (20.5)	0.676
Chronic kidney disease	55 (33.3)	20 (37.7)	35 (31.2)	0.480
Dialysis	41 (24.8)	15 (28.3)	26 (23.2)	0.563
Atrial fibrillation	15 (9.0)	5 (9.4)	10 (8.9)	1.000
Coronary artery disease	102 (61.8)	29 (54.7)	73 (65.1)	0.231
Myocardial infarction	10 (6.0)	3 (5.6)	7 (6.2)	1.000
PTCA	24 (14.5)	6 (11.3)	18 (16.0)	0.486
CABG	10 (6.0)	1 (1.8)	9 (8.0)	0.170
Smoking	66 (40.0)	21 (39.6)	45 (40.1)	1.000
Current smokers	35 (21.2)	13 (24.5)	22 (19.6)	0.542
Alcoholic	49 (29.6)	17 (32.0)	32 (28.5)	0.716
Current alcoholics	29 (17.5)	11 (20.7)	18 (16.0)	0.513
Laboratory findings				
Fasting glucose (mg/dL)	144.2 ± 67.2	150.2 ± 71.9	141.2 ± 65.0	0.520
Hemoglobin A1c (%)	7.4 ± 1.4	7.3 ± 1.3	7.5 ± 1.4	0.318
Total cholesterol (mg/dL)	147.6 ± 44.7	146.6 ± 48.5	148.1 ± 42.9	0.842
Triglycerides (mg/dL)	129.6 ± 100.5	132.0 ± 53.0	128.4 ± 124.1	0.840
HDL cholesterol (mg/dL)	36.3 ± 11.5	35.9 ± 12.3	36.6 ± 11.2	0.725
LDL cholesterol (mg/dL)	90.0 ± 37.3	89.0 ± 38.2	90.5 ± 36.9	0.819
hsCRP (mg/L)	20.3 ± 38.6	25.3 ± 37.5	18.0 ± 39.1	0.439
Albumin (g/dL)	3.8 ± 1.6	4.1 ± 4.0	3.6 ± 0.5	0.400
Uric acid (mg/dL)	5.5 ± 1.8	5.6 ± 1.6	5.4 ± 1.8	0.559
Creatinine (mg/dL)	2.6 ± 3.1	2.8 ± 3.1	2.5 ± 3.0	0.595
Magnesium (mEq/L)	1.7 ± 0.2	1.7 ± 0.2	1.7 ± 0.3	0.615

Values are mean ± SD (range). *HTN* indicates hypertension group, *Pts* patients, *AF* atrial fibrillation, *PTCA* percutaneous transluminal coronary angioplasty, *HDL* high dense lipoprotein, *LDL* low dense lipoprotein, *hs-CRP* high sensitive C-reactive protein

Coronary angiographic and clinical parameters were shown in Table 2. There was no difference in coronary angiographic characteristics between the two groups.

**Table 2:** Coronary angiographic and clinical parameters according to blood pressure control

Coronary artery disease	Total (*n* = 165 Pts) (*n* = 201 Limb) (*n* = 246 Lesion)	Controlled HTN (*n* = 53 Pts) (*n* = 66 Limb) (*n* = 90 Lesion)	Uncontrolled HTN (*n* = 112 Pts) (*n* = 135 Limb) (*n* = 156 Lesion)	*P* Value
Stents implantation	57 (34.5)	20 (37.7)	37 (33.0)	0.601
Routine CAG	138 (83.6)	31 (77.5)	107 (85.6)	0.226
Lesion site				
Left main	13 (9.4)	3 (6.8)	10 (10.6)	0.550
Left artery descending artery	59 (42.7)	19 (43.1)	40 (42.5)	1.000
Left circumflex artery	49 (35.5)	16 (36.3)	33 (35.1)	1.000
Right coronary artery	49 (35.5)	14 (31.8)	35 (37.2)	0.572
Multi-vessel disease	56 (33.9)	19 (35.8)	37 (33.0)	0.728
1VD	41 (24.8)	9 (16.9)	32 (28.5)	
2VD	34 (20.6)	13 (24.5)	21 (18.7)	
3VD	22 (13.3)	6 (11.3)	16 (14.2)	
CTO lesion	23 (13.9)	6 (11.3)	17 (15.1)	0.633
Onsite elective PCI	43 (26.0)	16 (30.1)	27 (24.1)	0.450

Values are mean ± SD (range). *HTN* indicates hypertension group, *Pts* patients, *CAG* coronary angiography, *1VD* 1 vessel disease, *2VD* 2 vessel disease, *3VD* 3 vessel disease, *CTO* chronic total occlusion, *PCI* percutaneous coronary intervention

The frequencies of beta blockers (BB), angiotensin converting enzyme inhibitor (ACEi), angiotensin receptor blocker (ARB), statins and antiplatelet drugs were similar between the two groups on admission and 30 days after BTK interventions in Table 3.

**Table 3:** Medications according to blood pressure control on admission and 30 days after BTK intervention

Variable. n%	Total (*n* = 165 Pts) (*n* = 201 Limb) (*n* = 246 Lesion)	Controlled HTN (*n* = 53 Pts) (*n* = 66 Limb) (*n* = 90 Lesion)	Uncontrolled HTN (*n* = 112 Pts) (*n* = 135 Limb) (*n* = 156 Lesion)	*P* Value
In-hospital medications				
Aspirin	165 (100.0)	53 (100.0)	112 (100.0)	1.000
Clopidogrel	152 (92.1)	48 (90.5)	104 (92.8)	0.758
Cilostazol	81 (49)	28 (52.8)	53 (47.3)	0.617
Warfarin	12 (7.2)	3 (5.6)	9 (8.0)	0.753
Sarpogrelate	61 (36.9)	21 (39.6)	40 (35.7)	0.730
Diuretics	41 (24.8)	11 (20.7)	30 (26.7)	0.446
ß-blockers	58 (35.1)	21 (39.6)	37 (33)	0.485
Ca-blockers	84 (50.9)	29 (54.7)	55 (49.1)	0.510
ACE-inhibitors	36 (21.8)	10 (18.8)	26 (23.2)	0.687
ARBs	73 (44.2)	25 (47.1)	48 (42.8)	0.618
Statins	114 (69)	39 (73.5)	75 (66.9)	0.472
30 days medications				
Aspirin	155 (93.9)	52 (98.1)	103 (91.9)	0.170
Clopidogrel	140 (84.8)	48 (90.5)	92 (82.1)	0.244
Cilostazol	54 (32.7)	20 (37.7)	34 (30.3)	0.377
Warfarin	10 (6.0)	3 (5.6)	7 (6.2)	1.000
Sarpogrelate	60 (36.3)	21 (39.6)	39 (34.8)	0.605

Values are mean ± SD (range). *BTK* indicates Below-the-knee, *HTN* hypertension, *Pts* patients, *ACE* angiotensin converting enzyme, *ARB* angiotensin II receptor blocker

The procedural and peripheral angiographic characteristics at baseline are shown in the Table 4. The most frequently treated vessel was the anterior tibial artery. The incidences of severe calcification, chronic total occlusion (CTO) and proximal lesions were higher in the uncontrolled HTN group. However, Rutherford classifications, ABI, lesion types, lesions length and procedural approach were similar between the two groups. Technical and clinical successes were obtained in 94 % of all patients.

**Table 4:** Peripheral angiographic and clinical parameters of target lesions according to blood pressure control

Variable. n%	Total (*n* = 165 Pts) (*n* = 201 Limb) (*n* = 246 Lesion)	Controlled HTN (*n* = 53 Pts) (*n* = 66 Limb) (*n* = 90 Lesion)	Uncontrolled HTN (*n* = 112 Pts) (*n* = 135 Limb) (*n* = 156 Lesion)	*P* Value
Ankle-brachial index	0.65 ± 0.4	0.72 ± 0.48	0.62 ± 0.4	0.159
Rutherford classifications (Limb)	5.01 ± 1.48	5.12 ± 1.41	4.95 ± 1.52	0.453
Limb site				
Right	108 (65.4)	35 (66.0)	73 (65.1)	1.000
Left	93 (56.3)	31 (58.4)	62 (55.3)	0.739
Both	36 (21.8)	13 (24.5)	23 (20.5)	0.552
Lesion locations				
Above the knee - Pts	82 (49.6)	25 (47.1)	57 (50.8)	0.739
Above the knee - Limb	88 (43.7)	26 (39.3)	62 (45.9)	0.450
lliac - Pts	10 (6.0)	4 (7.5)	6 (5.3)	0.728
lliac - Limb	10 (4.9)	4 (6.0)	6 (4.4)	0.732
Femoral - Pts	77 (46.6)	24 (45.2)	53 (47.3)	0.868
Femoral - Limb	83 (41.2)	25 (37.8)	58 (42.9)	0.543
Popliteal - Pts	20 (12.1)	5 (9.4)	15 (13.3)	0.612
Popliteal - Limb	21 (10.4)	6 (9.0)	15 (11.1)	0.808
Below the knee - Pts				
Below the knee - Limb				
Tibial - Pts	156 (94.5)	49 (92.4)	107 (95.5)	0.470
Tibial - Limb	183 (91.0)	57 (86.3)	126 (93.3)	0.119
ATA - Pts	119 (72.1)	37 (69.8)	82 (73.2)	0.711
ATA - Limb	140 (69.6)	43 (65.1)	97 (71.8)	0.333
PTA - Pts	74 (44.8)	25 (47.1)	49 (43.7)	0.739
PTA - Limb	76 (37.8)	26 (39.3)	50 (37.0)	0.759
Peroneal - Pts	44 (26.6)	16 (30.1)	28 (25.0)	0.572
Peroneal - Limb	47 (23.3)	17 (25.7)	30 (22.2)	0.598
Lesion site				
Proximal	151 (61.3)	45 (50.0)	106 (67.9)	0.007
Mid	30 (12.1)	15 (16.6)	15 (9.6)	0.110
Distal	24 (9.7)	12 (13.3)	12 (7.6)	0.182
Ostium	42 (17)	19 (21.1)	23 (14.7)	0.221
Lesion type				
Concentric	38 (15.4)	16 (17.7)	22 (14.1)	0.467
Eccentric	61 (24.7)	20 (22.2)	41 (26.2)	0.541
Total occlusion	147 (59.7)	54 (60.0)	93 (59.6)	1.000
Lesion characteristics				
CTO	119 (48.3)	35 (38.8)	84 (53.8)	0.025
Diffuse (≥2 cm)	231 (93.9)	83 (92.2)	148 (94.8)	0.418
Calcification	119 (48.3)	36 (40.0)	83 (53.2)	0.048
Procedure				
SubIntimal approach	57 (23.1)	23 (25.5)	34 (21.7)	0.532
POBA	228 (92.6)	84 (93.3)	144 (92.3)	1.000
Stent type	18 (7.3)	6 (6.6)	12 (7.6)	1.000
Smart control	1 (0.4)	1 (1.1)	0 (0.0)	
Xpert	14 (5.6)	3 (3.3)	11 (7.0)	
Chromis deep	2 (0.8)	2 (2.2)	0 (0.0)	
Maris deep	1 (0.4)	0 (0.0)	1 (0.6)	
Technical Success	230 (93.4)	82 (91.1)	148 (94.8)	0.287
Clinical Success	232 (94.3)	85 (94.4)	147 (94.2)	1.000

Values are mean ± SD (range). *HTN* indicates hypertension group, *Pts* patients, *ATA* anterior tibial artery, *PTA* posterior tibial artery, *CTO* chronic total occlusion, *POBA* plain old balloon angioplasty

Table 5 shows the procedural complications after BTK interventions. There was no difference in procedural complications including arterio-venous (AV) fistula, pseudo-aneurysm, access site hematoma, gastro-intestinal (GI) bleeding, blood transfusion rates, contrast induced nephropathy and arrhythmia between the two groups.

**Table 5:** Periprocedural complications according to blood pressure control

Type of complications	Total (*n* = 165 Pts) (*n* = 201 Limb) (*n* = 246 Lesion)	Controlled HTN (*n* = 53 Pts) (*n* = 66 Limb) (*n* = 90 Lesion)	Uncontrolled HTN (*n* = 112 Pts) (*n* = 135 Limb) (*n* = 156 Lesion)	*P* Value
AV fistula	1 (0.6)	1 (1.8)	0 (0.0)	0.321
Pseudo-aneurysm	2 (1.2)	1 (1.8)	1 (0.8)	0.541
Access site hematoma				
minor (<4 cm)	4 (2.4)	1 (1.8)	3 (2.6)	1.000
Major (≥4 cm)	14 (8.4)	4 (7.5)	10 (8.9)	1.000
G.I. bleeding	4 (2.4)	0 (0.0)	4 (3.5)	0.307
Transfusion	91 (55.1)	31 (58.4)	60 (53.5)	0.617
Transfusion (Unit)	5.81 ± 10.4	6.9 ± 11.2	5.2 ± 10.0	0.325
Acute renal failure	4 (2.4)	1 (1.8)	3 (2.6)	1.000
Congestive heart failure	3 (1.8)	1 (1.8)	2 (1.7)	1.000
Arrhythmia	4 (2.4)	2 (3.7)	2 (1.7)	0.594

Values are mean ± SD (range). *HTN* indicates hypertension group, *Pts* patients, *AV* arterio-venous, *G.I.* gastrointestinal

Clinical and peripheral angiographic data at 12 months are presented in Table 6. There was no difference in the incidence of mortality, myocardial infarction, cerebrovascular infarction in both groups. The incidences of TLR, target extremity revascularization (TER), limb salvage rate, binary restenosis, primary and secondary patency were also similar between the two groups at 12 months. And there was no patient who refused the amputation, if the patients were indicated. However, all patients with HTN (*n* = 165) had higher incidence of MALE compared to those without HTN (*n* = 15) at 12 month follow-up (37.6 % vs. 6.7 %, *P* = 0.021).

**Table 6:** Clinical outcomes following BTK interventions in patients with CLI according to blood pressure control at 12 months

Variable. n%	Total (*n* = 165 Pts) (*n* = 201 Limb) (*n* = 246 Lesion)	Controlled HTN (*n* = 53 Pts) (*n* = 66 Limb) (*n* = 90 Lesion)	Uncontrolled HTN (*n* = 112 Pts) (*n* = 135 Limb) (*n* = 156 Lesion)	*P* Value
12-months clinical outcomes				
Mortality	9 (5.4)	2 (3.7)	7 (6.2)	0.720
Cardiac death	5 (3.0)	1 (1.8)	4 (3.5)	1.000
TLR, Pts	18 (10.9)	6 (11.3)	12 (10.7)	0.907
TLR, Limb	21 (10.4)	6 (9)	15 (11.1)	0.808
TER, Pts	20 (12.1)	7 (13.2)	13 (11.6)	0.769
TER, Limb	25 (12.4)	7 (10.6)	18 (13.3)	0.655
Non TER	7 (4.2)	3 (5.7)	4 (3.6)	0.534
Amputations - Pts	40 (24.2)	18 (33.9)	22 (19.6)	0.045
Amputations - Limb	41 (20.3)	19 (28.7)	22 (16.2)	0.061
Major				
above the knee	0 (0.0)	0 (0.0)	0 (0.0)	1.000
above the ankle - Pts	10 (6.0)	7 (13.2)	3 (2.6)	0.013
above the ankle - Limb	10 (4.9)	7 (10.6)	3 (2.2)	0.016
minor (below the ankle) - Pts	30 (18.1)	11 (20.7)	19 (16.9)	0.556
minor (below the ankle) - Limb	31 (15.4)	12 (18.1)	19 (14)	0.533
Myocardial infarction	2 (1.2)	1 (1.8)	1 (0.8)	0.541
PTCA	7 (4.2)	3 (5.6)	4 (3.5)	0.682
Cerebrovascular accidents	1 (0.6)	0 (0.0)	1 (0.8)	1.000
Limb salvage	147/156 (94.2)	45/51 (88.2)	102/105 (97.1)	0.059
Angiogram to follow-up	55 (33.3)	18 (33.9)	37 (33)	0.906
CT	21 (38.1)	8 (44.4)	13 (35.1)	0.505
PAG	42 (76.3)	13 (72.2)	29 (78.3)	0.738
Binary restenosis	35 (63.6)	11 (61.1)	24 (64.8)	0.786
Total re-occlusion	29 (52.7)	8 (44.4)	21 (56.7)	0.391
Primary patency	20 (36.3)	7 (38.8)	13 (35.1)	0.786
Secondary patency	39 (70.9)	11 (61.1)	28 (75.6)	0.264
Non-Total occlusion; Runoff (≥1) included Collateral, to distal	53 (96.3)	17 (94.4)	36 (97.2)	1.000

Values are mean ± SD (range). *BTK* indicates Below-the-knee artery, *CLI* critical limb ischemia, *HTN* hypertension, *Pts* patients, *TLR* target lesion revascularization, *TER* target extremity revascularization, *PTCA* percutaneous transluminal coronary angioplasty, *CT* computed tomography, *PAG* follow-up invasive peripheral angiography

In univariate analysis, HTN, DM foot, regional wall motion abnormality of LV, mitral valve calcification, congestive heart failure, chronic kidney disease and dialysis were significantly associated with MALE in patients with CLI after peripheral angioplasty at 12 months. In multivariate analysis, HTN (*P* = 0.002), mitral valve calcification (*P* = 0.040) and dialysis (*P* = 0.029) were independent risk factors for MALE (Table 7) at 12 months.

**Table 7:** Univariate and multivariate Cox analyses for MALE in patients with CLI after peripheral angioplasty at 12-month follow-up

	Univariate analysis		Multivariate analysis	
Variable. N (%)	OR (95 % CI)	*P*-Value	OR (95 % C.I)	*P*-Value
Hypertension (itself)	2.093 (1.106 – 4.098)	0.017	3.867 (1.625 – 9.199)	0.002
DM foot	1.903 (0.935 – 3.876)	0.076		
RWMA	2.342 (1.190 – 4.608)	0.014		
Mitral valve calcification	3.029 (1.297 – 7.073)	0.010	2.915 (1.048 – 8.107)	0.040
CHF	2.342 (1.190 – 4.608)	0.014		
CKD	1.966 (1.049 – 3.685)	0.035		
Dialysis	3.000 (1.496 – 6.014)	0.002	5.221 (1.184 – 23.02)	0.029

*OR* odds ratio, *CI* confidence interval, *DM* diabetes mellitus, *RWMA* reginonal wall motion abnormality, *CHF* congestive heart failure-systolic, *CKD* chronic kidney disease

**Fig. 2:**
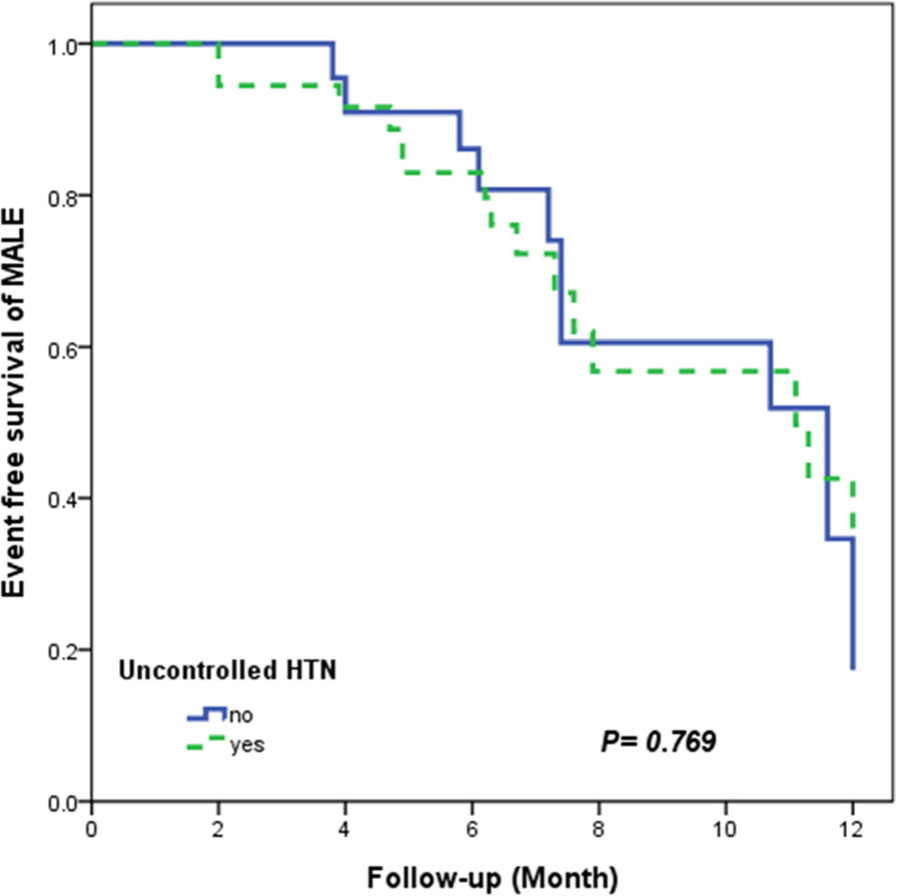
Kaplan-Meier analysis for survival free from MALE in both study groups

Kaplan-Meier curves showed that event free survivals of MALE are similar in both groups at 12 month follow-up (*P* = 0.456; Fig. 2).

## Discussion

The main findings of the present study are that despite the patients with uncontrolled HTN had more proximal target lesions, higher incidences of CTO and severe calcifications, however, at 12 month follow-up, there was no difference in the incidence of mortality, TLR, TER, limb salvage rate in both groups following successful BTK endovascular revascularization. Rather, the amputation rate was higher in patients with controlled HTN compared to that of uncontrolled HTN. Therefore, it is difficult to interpret the difference in the results of the impact of BP control on mid-term clinical outcomes in hypertensive patients with CLI following BTK interventions. However, HTN itself was an independent risk factor for MALE in hypertensive CLI patients with BTK lesions, suggesting more intensive medical therapy with close clinical follow up will be required for all patients with HTN in real world clinical practice.

Previous study reported that the prevalence of PAD in patients with HTN is higher than those without HTN.[4] Essential HTN is associated with impaired regulation of vascular tone and endothelial dysfunction in the peripheral artery [9]. Evidence from these studies suggests that acetylcholine mediated as well as flow-mediated dilation is impaired in essential HTN and that the dysfunction in part is related to defects in the nitric oxide system [10, 11].

Guidelines published for the detection and treatment of HTN (The joint National Committee on Prevention, Detection, Evaluation and Treatment of high Blood Pressure, and European Society of Cardiology) recommend PAD as evidence of clinical cardiovascular disease. Hypertensive patients with PAD need drug therapy no matter which stages of hypertension they are in [12]. However, due to the lack of specific PAD symptoms, clinical awareness of PAD is very low in the primary care setting, which translates into missed opportunities to treatment of HTN in hypertensive patients with PAD. Even though the hypertensive patients knew that they had high blood pressure, 30 ~ 50 % of those with HTN are either untreated or undertreated [8].

Previous meta-analyses of randomized placebo-controlled trials indicate that antihypertensive therapy in patients with uncontrolled HTN reduces the risk of major cardiovascular adverse events (stroke by 30 %, coronary heart disease by 10 % to 20 %, congestive heart failure by 40 %, and total mortality by 10 %) [13], which can be conversely interpreted that uncontrolled HTN can be very important risk factor for cardiovascular adverse events. Although, it remains controversial whether the risk of cardiovascular events is related solely to the blood pressure achieved or also to the manner in which it is achieved [7]. And recent study also reported that the risk of PAD was increased with increasing HTN grade (HTN grade 3, OR 1.62, *P* = 0.006) [14], and recent national review reported that the hypertensive patients with major lower extremity amputations had higher incidence of below the knee amputations than above the knee amputations, which is suggesting that HTN can affect smaller and more distal vessels of lower extremities [15].

Therefore, we hypothesized that uncontrolled HTN also can affect the mid or long term clinical outcomes after BTK interventions in hypertensive patients with CLI. This is the first study to compare the 12 months clinical outcomes after BTK interventions in hypertensive patients with according BP control.

CLI represents the most severe stage of peripheral vascular disease, with complications of limb loss [16, 17]. The clinical presentations range from rest pain, ischemic ulcers to gangrene. Apart from the potential loss of limb which is usually evident at presentation, the co-existent cardiovascular morbidity and mortality presents an even greater threat. Previous longitudinal follow-up studies have been shown that HTN is amongst the most important risk factors for PAD along with increasing age, smoking, DM and dyslipidemia [18–20].

In this study, the peripheral angiographic characteristics at baseline showed that the incidences of severe calcification, CTO and proximal lesions were higher in the uncontrolled HTN group, which is consistent with the previous reports that severe HTN was more strongly associated with proximal disease [21], and the most important factor influencing the progression of atherosclerosis [22].

However, in this study, there was no difference in the incidence of mortality, TLR, TER, limb salvage rate in both groups at 12 months. Rather, amputation rate was higher in patients with controlled HTN compared to those with uncontrolled HTN (*P* = 0.045). Conversely, the patients with uncontrolled HTN had a trend of higher limb salvage rate than those with controlled HTN (*P* = 0.059). Therefore, it is difficult to interpret this result. Our speculation and interpretations includes 1) differences in amputation rates might be ‘by chance’ due to relatively small number of study population, 2) significant proportion of the controlled HTN pts might be associated with longer history of hypertension, causing higher chance of advanced atherosclerosis and subsequent end organ damage, 3) despite of successful BTK intervention, uncontrolled HTN group might have more advanced wound condition that cannot be adjusted at the time of presentation due to longer history of atherosclerotic vascular disease.

Among those with CLI enrolled in this study, there were common peripheral angiographic features regardless of BP control in both groups. More than half patients had above the knee lesions, more proximal lesion sites, total occlusion type of lesions in both groups and most of the patients (>90 %) had diffuse long lesions (≥2 cm), which are consistent with the characteristics of unrecognized lower extremity peripheral artery disease in hypertensive adults [14]. And in this study, all patients with HTN (*n* = 165) had higher incidence of MALE compared to those without HTN (*n* = 15) at 12 month follow-up (37.6 % vs. 6.7 %, *P* = 0.021). In this regard, we can postulate that HTN itself is an independent risk factor for BTK lesions in patients with CLI, regardless of BP control.

Increased pulse pressure (PP) is known to be associated with arterial stiffness, leading to increased arterial pulse wave velocity. This causes a faster reflection of systolic pulse waves from the peripheral artery and causes a boost to late systolic BP, a greater fall in pressure in diastole and an increased PP [23]. In hypertensive patients, arterial compliance was reduced already with borderline PAD and increasing arterial stiffness plays a major pathophysiological role in the development of both increased PP and atherosclerotic lesions in the peripheral arteries [24]. In this study, the patients with uncontrolled HTN had increased PP compared to those with controlled HTN (mean PP; 83.8 ± 42.0 vs. 57.7 ± 0.8 mmHg, *P* < 0.001) as expected. However, there was no significant correlation between PP and 12-month clinical outcomes after BTK interventions in hypertensive patients with CLI according to BP control (*P* = 0.497).

### Study limitation

This study has several obvious limitations. First, we used retrospective analysis, although this study was performed as a prospective study. However, this study results is still meaningful due to the nature of study. We cannot perform randomized clinical trial with this exact title in terms of ethical issues.

Second, Uncontrolled HTN was defined as a blood pressure of > 140 mmHg systolic and 90 mmHg diastolic under anti-hypertensive treatment including patients who lack blood pressure control secondary to poor adherence and/or an inadequate treatment regimen, as well as those with true treatment resistance, which can be limitations due to the difference of pathophysiology between inadequate treatment and true treatment resistance. However, in this study, we showed that regardless of blood pressure control, HTN itself was an independent risk factor for MALE in CLI patients with BTK lesions. Third, there are small numbers of patients without HTN, which could be too small to compare with those with HTN. However, this study is retrospective observational study for the patients who underwent the peripheral angioplasties. Therefore, we could not enroll the patients without hypertension as a control group. Further a prospective study should be considered to get final conclusion.

Fourth, similar to many other trials in interventional cardiology, this was not a blinded study. In addition, patients were enrolled only in a single, high volume center that might have unique patient referral pattern and interventional technique. Fifth, even though we minimized the confounding effects from the baseline biases with multivariate logistic analysis, it is possible that some potential confounders might have crept in. Sixth, this study had no financial support, and no external angiography was available for adjudication of the end points. However, the size of the observed effect of uncontrolled HTN in the BTK lesions leaves few chances for these results to be controverted in a multicenter, randomized study. Finally, clinical results achieved by an integrated multidisciplinary approach to CLI in well-organized specialized center may not be reproduced in patients with uncontrolled HTN in other centers with different organization.

## Conclusions

Even though the patients with uncontrolled HTN had more proximal target lesions, higher incidences of CTO and severe calcifications, there was no difference in the incidence of major adverse events following BTK interventions in both groups at 12 months. Rather, the amputation rates were higher in patients with controlled HTN compared to those of uncontrolled HTN and there were common peripheral angiographic features regardless of BP control in both groups. Regardless of blood pressure control, HTN itself was an independent risk factor for MALE in CLI patients with BTK lesions, suggesting more intensive medical therapy with close clinical follow up will be required in real world clinical practice.

## Consent

Written informed consent was obtained from the patient for the publication of this report and any accompanying images.
